# Safety and efficacy of tixagevimab/cilgavimab for pre-exposure prophylaxis in kidney transplant recipients: a multicenter retrospective cohort study

**DOI:** 10.1007/s40620-024-01889-9

**Published:** 2024-05-23

**Authors:** Simona Simone, Virginia Pronzo, Francesco Pesce, Davide Fiore Bavaro, Barbara Infante, Silvia Mercuri, Annalisa Schirinzi, Antonella Panaro, Eleonora Conte, Alessandra Belati, Dario Troise, Paola Pontrelli, Francesca Conserva, Pasquale Gallo, Maddalena Panico, Marco Spilotros, Giuseppe Lucarelli, Annalisa Saracino, Giovanni Stallone, Francesca Di Serio, Pasquale Ditonno, Loreto Gesualdo

**Affiliations:** 1 Nephrology, Dialysis and Transplantation Unit, Department of Emergency and Organ Transplantation, University of Bari Aldo Moro, Bari, Italy; 2 Department of Biomedical Sciences and Human Oncology, Clinic of Infectious Diseases, University of Bari Aldo Moro, Bari, Italy; 3 Clinic Pathology Unit, University Hospital of Bari, Bari, Italy; 4 Renal Unit, Department of Medical and Surgical Sciences, University of Foggia, Foggia, Italy; 5 Urology, Andrology and Kidney Transplantation Unit, Department of Emergency and Organ Transplantation, University of Bari “Aldo Moro”, 70124 Bari, Italy

**Keywords:** SARS-CoV-2, Monoclonal antibodies, Kidney transplant, Prophylaxis

## Abstract

**Background:**

Immunocompromised patients show an impaired vaccine response and remain at high risk of severe COVID-19, despite vaccination. Neutralizing monoclonal antibodies against severe acute respiratory syndrome coronavirus 2 (SARS-CoV-2) have been developed for prophylaxis and treatment. The combination tixagevimab/cilgavimab (AZD7442) has been authorized for emergency use as pre-exposure prophylaxis for COVID-19, but data on safety and efficacy in kidney transplant recipients during the Omicron period are limited.

**Methods:**

We conducted a multicenter retrospective cohort study including 253 kidney transplant recipients, of whom 98 were treated with tixagevimab/cilgavimab 150 mg/150 mg and 155 who received only four doses of the BNT162b2 mRNA vaccine.

**Results:**

Only 13.3% of patients developed SARS-CoV-2 infection after the administration of tixagevimab/cilgavimab; in comparison, 34.2% of patients had been infected after the fourth dose of vaccine (*p* = 0.00013). Most infected patients in the AZD7442 group remained asymptomatic (92.3% vs 54.7%), 7.7% had mild symptoms and none had severe disease, need for hospitalization or died, while in the control group, 9.4% of patients had moderate or severe disease (*p* = 0.04). Using Kaplan–Meier curves we demonstrated that the controls presented early infection compared to the AZD7442 group (*p* = 0.000014). No changes in eGFR or proteinuria, assessed before and after the administration, were observed.

**Conclusions:**

In conclusion, our study showed that tixagevimab/cilgavimab 150/150 mg is effective and safe in preventing infection and severe disease when administered to patients with weak or no response to COVID-19 vaccine.

**Graphical abstract:**

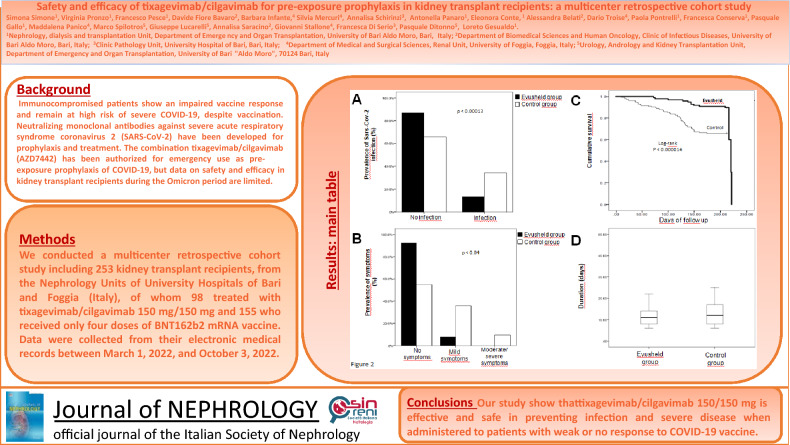

## Introduction

The impact of the COVID-19 pandemic on solid organ transplant recipients and on transplantation activity has been highly negative. Indeed, solid organ transplant recipients with severe acute respiratory syndrome coronavirus 2 (SARS-CoV-2) infection are at higher risk of severe disease and poor outcome [1]. Immunosuppressive therapies and co-morbidities are responsible for the greater susceptibility of solid organ transplant recipients in developing more severe forms of COVID-19, who are burdened by higher mortality rate and intensive care unit admission compared to the general population and other patients.

Several studies have shown the safety of mRNA vaccines in immunocompromised patients who were often excluded from clinical trials of COVID-19 vaccination, showing that the rate of seroconversion was lower than in healthy controls [2, 3]. The decreased immunogenicity of a two-dose regimen vaccination against SARS-CoV-2 in patients with impaired immune response, likewise with solid organ transplant recipients and dialysis patients [4, 5], prompted authorities to approve the administration of additional doses of vaccine in these subjects. A regimen of three doses of mRNA COVID-19 vaccine in a large cohort of kidney transplant recipients (KTRs) has been demonstrated to induce a late but effective immune response against SARS-CoV-2 infection, reducing morbidity and mortality in this population [6].

In addition to vaccination, several drugs have been tested in terms of safety and efficacy for the treatment and prophylaxis of COVID-19. Regardless of the type of intervention, therapeutic or prophylactic, timing is crucial to achieve optimal results.

Though vaccination must be considered the first line of intervention in SARS-CoV-2 infection, certain groups of individuals may not have an adequate antibody response to COVID-19 vaccines and others may not complete the vaccination schedule due to documented adverse events.

On December 8, 2021, AstraZeneca received Emergency Use Authorization (EUA), from the Food and Drug Administration (FDA), for the anti-SARS-CoV-2 monoclonal antibody combination AZD7442, to be used as pre-exposure prophylaxis for COVID-19. AZD7442 consists of two long-acting antibodies, tixagevimab (AZD8895) and cilgavimab (AZD1061), and can be administered to certain groups of adults and pediatric subjects (≥ 12 years old, weighing at least 40 kg), who are not currently infected with SARS-CoV-2. They bind the portion of the SARS-CoV-2 spike protein responsible for the interaction with the angiotensin converting enzyme-2 receptor of human cells. [7].

The results of PROVENT, a large randomized, double-blind, placebo-controlled, multi-center phase 3 trial, showed that a single intramuscular administration of AZD7442 (150 mg each of tixagevimab and cilgavimab) reduced the risk of developing symptomatic COVID-19 by 82.8% compared to placebo. Pharmacokinetic data showed that this combined treatment has an extended half-life, and serum levels remain elevated for 6 months after a single administration. The neutralizing activity of AZD7442, observed in the PROVENT study was mainly against beta, alpha and delta SARS-CoV-2 variants [8, 9].

In vitro studies have shown some neutralizing activity also against the Omicron BA.1 subvariant. However, further evidence has recently suggested that AZD7442 may be less effective against the Omicron strain [10].

Moreover, data on safety and efficacy of pre-exposure prophylaxis with AZD7442 in KTRs during the Omicron period are limited.

The aims of this real-world setting study were: (a) to evaluate the efficacy and safety of early pre-exposure prophylaxis with tixagevimab/cilgavimab, during the Omicron period in a cohort of KTRs, including patients who did not develop an adequate antibody response after three doses of BNT162b2 mRNA vaccine and patients who received a kidney transplant less than a year before; (b) to compare the former group with a cohort of KTRs who received four doses of BNT162b2 mRNA vaccine without pre-exposure prophylaxis.

## Materials and methods

### Study design and population

To assess the safety and efficacy of tixagevimab/cilgavimab in a cohort of KTRs, we conducted a multicenter observational retrospective cohort study of White adult KTRs receiving pre-exposure prophylaxis with tixagvimab/cilgavimab at the Nephrology Units of the University Hospitals of Bari and Foggia (Italy). A group of KTRs, vaccinated with four doses of BNT162b2 mRNA vaccine during the same period but who did not receive pre-exposure prophylaxis, was used for comparison. Data were collected from their electronic medical records between March 1, 2022, and October 3, 2022.

Tixagevimab/cilgavimab was administered to 57 KTRs who had shown a reduced response to a three-dose regimen of mRNA COVID-19 vaccine (defined as an anti-SARS-CoV-2 spike protein level < 0.80 U/mL). In this population, the antibody titer after three doses of mRNA vaccine was available prior to tixagevimab/cilgavimab administration. Forty-one KTRs who had received a kidney transplant less than 1 year before, received pre-exposure prophylaxis regardless of serological status. In this population of 41 patients, 13 received 4 doses of mRNA vaccine, 24 received 3 doses, and 4 received 2 doses of mRNA COVID-19 vaccine. Patients who tested negative for SARS-CoV-2, received a single intramuscular injection of AZD7442 (tixagevimab 150 mg + cilgavimab 150 mg) according to the manufacturer’s recommendations and the FDA indications at the time of writing. In this group, anti-receptor binding domain level was tested on blood samples collected before the administration of AZD7442 (T0EV) and one month after the administration (T1EV).

During the study period, the Omicron variant (sublineages BA.2, BA.4, BA.5) was predominant in Italy, with an estimated prevalence in April of 100% as reported by the Italian National Institute of Health [11]. The primary study outcome was the development of SARS-CoV-2 infection, defined as a newly positive result on reverse-transcriptase-polymerase-chain-reaction (RT-PCR) or antigen test on nasal swabs in the follow-up period. Secondary outcomes included: severity of symptoms, hospitalization, or death from COVID-19 infection, changes in renal function and proteinuria, and the occurrence of adverse events after AZD7442 administration.

The AZD7442 group was followed up between the date of administration and the end of the study period while the control group was followed up between the date of the administration of the fourth vaccine dose and the end of the study period.

The Local Ethics Committees approved the study protocol (study n°6845, prot. n°0050551) and written informed consent was obtained before enrollment.

### Data collection

Data related to age, gender, ethnicity, body mass index (BMI), type of donor, time from kidney transplantation, immunosuppressive therapies at baseline, comorbidities, estimated glomerular filtration rate ([eGFR], calculated by Chronic Kidney Disease Epidemiology Collaboration [CKD-EPI] equation) and proteinuria (mg/24 h) before and after the administration of AZD7442 or fourth vaccine dose, COVID-19 vaccination status, and history, timing and symptoms of COVID-19 infection were retrieved for both groups from their electronic medical records.

Data on clinical manifestations and patient management were collected. The severity of COVID-19 infection was classified as follows: no symptoms; mild symptoms (including cough, fever, sore throat, asthenia, anorexia, nasal congestion, headache, muscle pain) without radiologic findings of pneumonia; moderate/severe symptoms (including dyspnea with or without need for oxygen with non-invasive or invasive ventilation, chest imaging showing bilateral pneumonia, shock, multi-organ failure, need for hospitalization) [Table 1].

**Table 1:** Clinical characteristics of SARS-CoV-2 infection

Type	Clinical characteristics
Asymptomatic	No clinical symptoms or radiologic findings
Mild	Mild clinical symptoms, such as cough, fever, sore throat, asthenia, anorexia, nasal congestion, headache, muscle pain without chest imaging findings
Moderate/severe	Moderate to severe symptoms such as dyspnea with or without need for oxygen with non-invasive or invasive ventilation, chest imaging showing bilateral pneumonia, shock, multi-organ failure, need for hospitalization

Data on the duration of SARS-CoV-2 infection and the development of adverse events for both groups were obtained from the electronic medical records of routine visits.

### SARS-CoV-2 antibody detection assay

Blood samples were collected in BD vacutainer SST II Advance serum collection tubes for each patient. Tubes were then centrifuged for 10 min at 3000*g* within 2 h of collection, and the resulting serum fraction was transferred to a clean Eppendorf tube and stored at − 80 °C until further use. Samples were tested on the commercially available Elecsys^®^ Anti-SARS-CoV-2 *S* assay (Roche Diagnostics International Ltd, Rotkreuz, Switzerland). The Elecsys Anti-SARS-CoV-2 S assay is a quantitative ECLIA that detects high-affinity antibodies to the SARS-CoV-2 S protein Receptor Binding Domain; it has a low risk of detecting weakly cross-reactive and unspecific antibodies. Results are automatically reported as the analyte concentration of each sample in U/mL, with < 0.80 U/mL interpreted as negative for anti-SARS-CoV-2 S antibodies and ≥ 0.80 U/mL interpreted as positive for anti-SARS-CoV-2 S antibodies.

The sensitivity and specificity of the enzyme immunoassay are excellent for detection of the antispike humoral response to SARS-CoV-2 infection (84% sensitivity and 100% specificity for Roche Elecsys) and are analogous to the antispike antibody assays used during immunogenicity assessments in mRNA vaccine clinical trials.

The assay was performed on specimens collected from all participants before (T0EV) and one month after (T1EV) the intramuscular administration of a single dose of tixagevimab/cilgavimab 150 mg/150 mg.

### Statistical analysis

Statistical analysis was performed using IBM SPSS statistics for Windows, version 25. Continuous variables were described as mean with standard deviation or median and interquartile range [25th; 75th percentiles], according to data distribution. Categorical variables are listed as counts or percentage. Differences between continuous variables were assessed using a Wilcoxon matched-pairs signed-rank test. Differences between proportions of categorical variables were assessed using Pearson’s Chi-squared test. Logistic regression was used to predict the category infection based on each clinical predictor. Kaplan–Meier curves were used to estimate the incidence of SARS-CoV-2 infection in the AZD7442 and control groups with differences assessed using the log-rank test. Statistical significance was considered if *p* values were < 0.05.

## Results

### Characteristics of the population

**Fig. 1:**
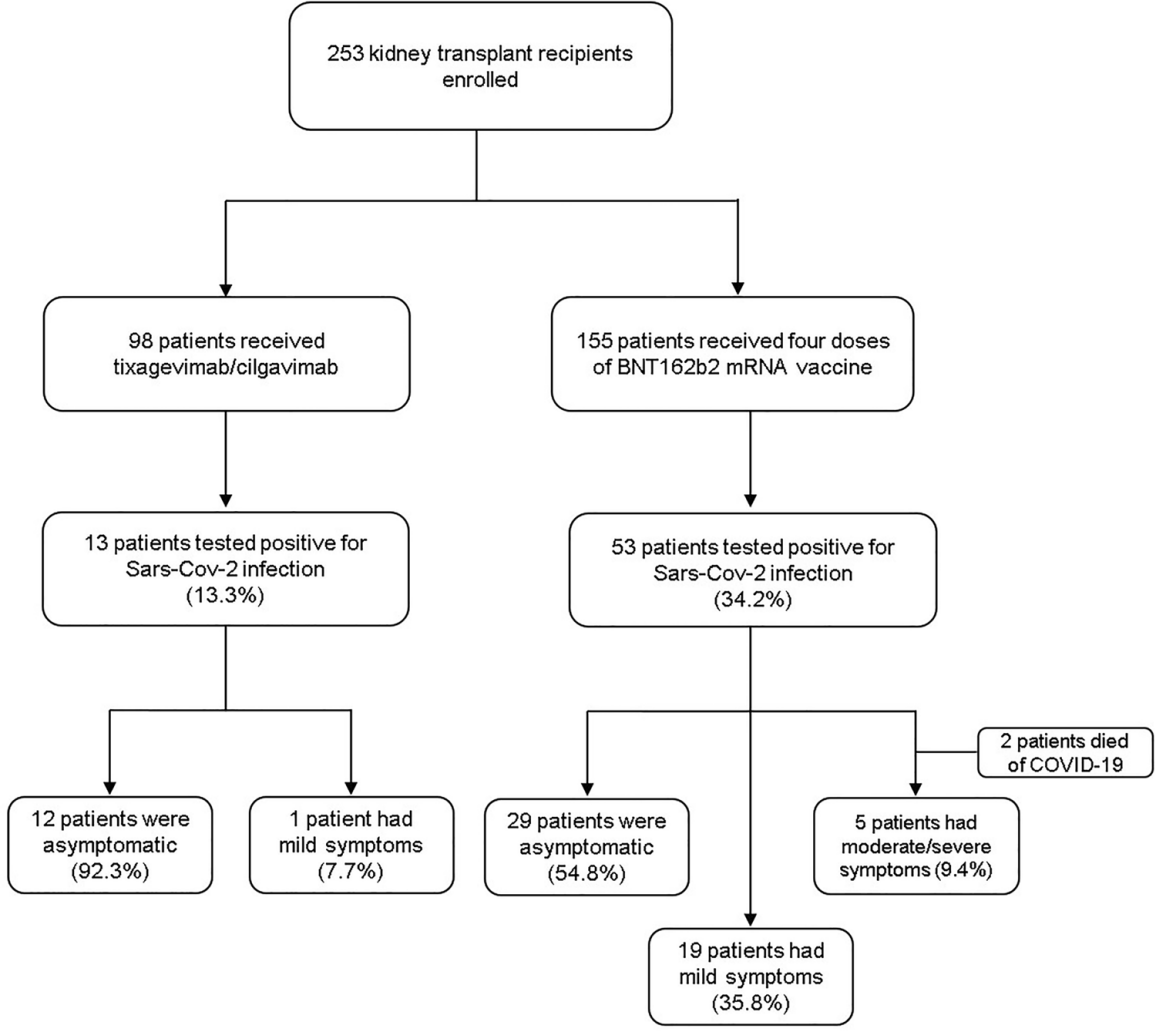
Flow chart with the number of patients included in the study, groups and follow-up

We included a total of 253 KTRs whose clinical and biological characteristics are reported in Table 2. The entire cohort was divided into two groups: the first group included 98 patients who received pre-exposure prophylaxis with a single intramuscular dose of tixagevimab/cilagvimab 150 mg/150 mg in accordance with the FDA and European Medicines Agency (EMA) regulations at the time of the study; the control group included 155 KTRs who received four doses of BNT162b2 mRNA vaccine but did not receive pre-exposure prophylaxis (Fig. 1).

**Table 2:** Patient characteristics

Variables	AZD7442 group (*n* = 98)	Control group (*n* = 155)
Age, year, median [IQR]	56 [48–64]	62 [52–69]
Sex, *n* [%]		
Male	60 [61.2]	100 [64.5]
Female	38 [38.8]	55 [35.5]
eGFR (mL/min/1.73 m^2^), median [IQR]	45.5 [36–60]	52 [39–69.5]
BMI, kg/m^2^, median [IQR]	25 [22–28]	25 [23–27.5]
Hypertension, *n* [%]	82 [83.7]	136 [87.7]
Diabetes, *n* [%]	16 [16.3]	24 [15.5]
Cardiovascular disease, *n* [%]	33 [33.7]	34 [21.9]
Liver disease, *n* [%]		
HBV-related	0 [0]	10 [6.5]
HCV-related	2 [2]	5 [3.2]
Malignancies, *n* [%]	9 [9.2]	23 [14.8]
History of SARS-CoV-2 infection, *n* [%]	17 [17.3]	26 [16.8]
Number of vaccines received, *n* [%]		
None	0 [0]	0 [0]
One	0 [0]	0 [0]
Two	4 [4.1]	0 [0]
Three	81 [82.6]	0 [0]
Four	13 [13.3]	155 [100]
Type of donor, *n* [%]		
Deceased	81 [82.7]	142 [91.6]
Living	17 [17.3]	13 [8.4]
Months from kidney transplantation, median [IQR]	14 [7–66]	106 [62.5–187.5]
Retransplantation, *n* [%]	13 [13.3]	14 [9.0]
Immunosuppressants, *n* [%]		
CNIs^a^	97 [99]	143 [92.3]
Antimetabolites^b^	81 [82.7]	115 [74.2]
Steroids	94 [95.9]	137 [88.4]
mTORi^c^	8 [8.2]	24 [15.5]

*BMI* body mass index, *eGFR* estimated Glomerular Filtration Rate (Chronic Kidney Disease Epidemiology Collaboration (CKD-EPI) equation), *CNIs* calcineurin inhibitors

^a^Includes tacrolimus or cyclosporine

^b^Includes mycophenolate mofetil, mycophenolic acid, or azathioprine

^c^mTORi mammalian target of rapamycin inhibitors including sirolimus and everolimus

In the AZD7442 group the median age was 56 years [IQR 48–64], 61.2% were male, and the median time from kidney transplantation to the end of the study period was 14 months [IQR 7–66]. Blood analysis at baseline revealed mean eGFR assessed by the CKD-EPI formula of 45.5 mL/min/1.73 m^2^ [IQR 36–60]. The standard immunosuppressive regimen consisted of steroids (95.9% of patients), calcineurin inhibitors including Tacrolimus and Cyclosporine (99% of patients), antimetabolites including mycophenolate mofetil, mycophenolic acid, or azathioprine (82.7% of patients), and mTORi (8.2% of patients). All KTR low or no responders to vaccine had been previously vaccinated with three doses of the BNT162b2—Pfizer vaccine. Among the KTRs who received tixagevimab/cilgavimab because they were less than one year post-transplant, 13 had been vaccinated with four doses, 24 with three doses and 4 with two doses of mRNA COVID-19 vaccine. There was a history of SARS-CoV-2 infection in 17.3% of subjects. None of them had previously received the casirivimab/imdevimab combination.

The control group was comparable in terms of clinical features, except for a longer time from transplantation (106 months, IQR 62.5–187.5 vs 14 [7–66]).

### Clinical outcomes and renal safety

**Fig. 2:**
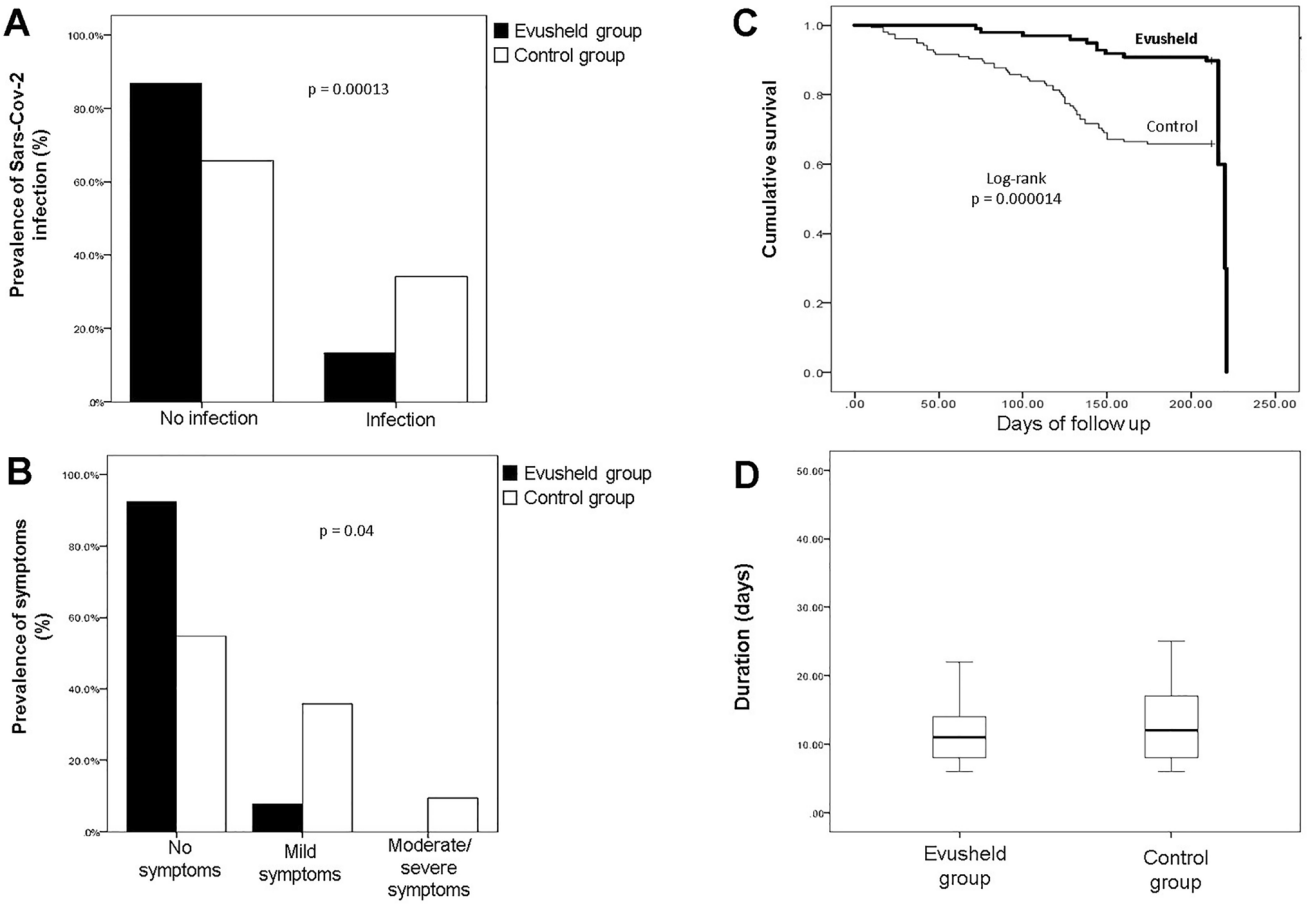
SARS-CoV-2 infection in kidney transplant recipients. **A** The panel shows a lower prevalence of infected patients within the AZD7442 group in comparison with the control group (*p* = 0.00013). **B** The prevalence of asymptomatic SARS-CoV-2 infection was significantly higher among patients treated with tixagevimab/cilgavimab than in the control group (*p* = 0.04). **C** Kaplan–Meier estimates of cumulative incidence of SARS-CoV-2 infection in the AZD7442 group (*n* = 98) and in the control group (*n* = 153) (*p* = 0.000014). **D** The figure shows that there was no significant difference in the duration of SARS-CoV-2 infection between the two groups

As of October 3, 2022, only 13.3% (13/98) of patients within the AZD7442 group tested positive for SARS-CoV-2 infection, whereas 86.7% of patients (85/98) did not develop infection. On the contrary, in the control group (KTRs vaccinated with a four-dose regimen of BNT162b2 mRNA vaccine), 34.2% (53/155) had been infected after the fourth dose of vaccine (*p* = 0.00013, Fig. 2A).

At univariate logistic regression analysis none of the clinical variables listed in Table 2 showed an impact on infection, while the administration of AZD7442 (*p* = 0.000357, OR = 0.294, 95% IC 0.150–0.576) and history of SARS-CoV-2 infection showed a protective effect (*p* = 0.005, OR = 0.175, 95% IC 0.052–0.587).

A higher proportion of infected patients in the AZD7442 group were asymptomatic compared to the 4-mRNA vaccine dose group (92.3% vs 54.7% *p* = 0.04, Fig. 2B). Only one (7.7%) patient in the AZD7442 group had mild symptoms, and no patients developed moderate/severe disease or need for hospitalization vs 5 patients (9.4%) in the 4-mRNA vaccine dose group who developed severe disease. The antimetabolite drugs were immediately withdrawn in all infected patients of both groups after diagnosis. No patients in the AZD7442 group were admitted to the intensive care unit or died during the study period. On the contrary, within the control group, two patients died of COVID-19-related complications.

Moreover, in order to estimate the cumulative incidence of SARS-CoV-2 infection and compare the timing of SARS-CoV-2 infection between the AZD7442 and control groups, Kaplan–Meier curves were used. The results, in Fig. 2C, show that the control group presented early infection compared to the AZD7442 group (*p* = 0.000014).

Moreover, in patients without pre-exposure prophylaxis, the duration of COVID-19 positivity was longer than in those who received tixagevimab/cilgavimab, but this difference was not statistically significant (Fig. 2D).

**Fig. 3:**
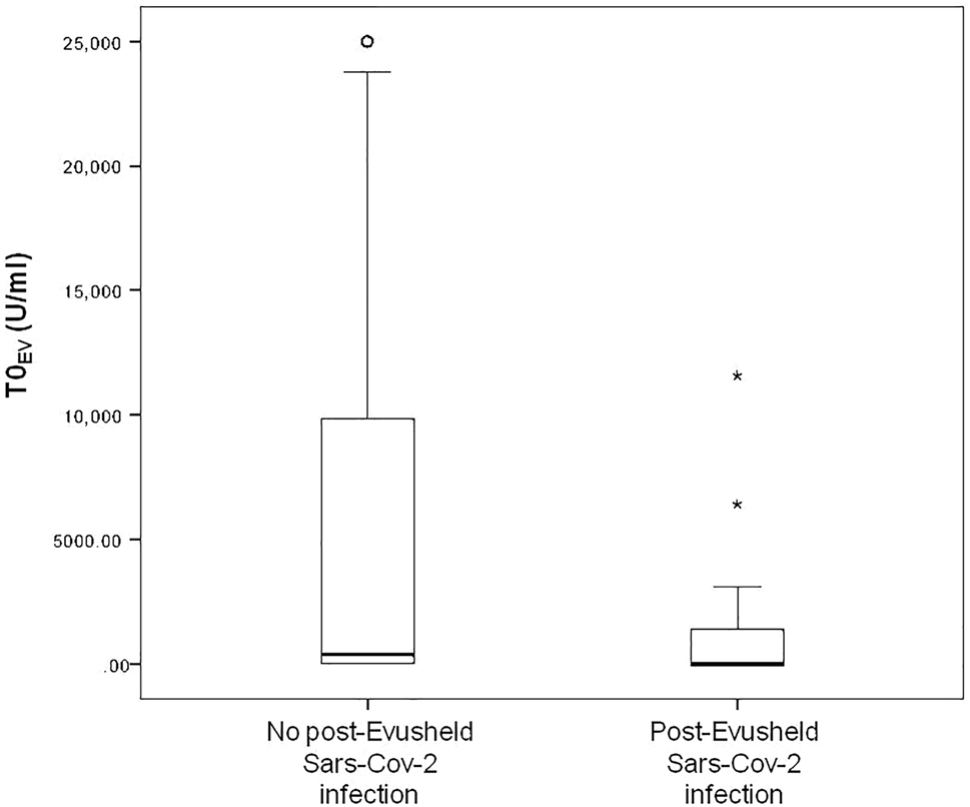
Comparison of SARS-CoV-2 anti-spike protein titer at T0EV between KTRs who developed COVID-19 infection to KTRs who did not. KTRs who tested positive for SARS-CoV-2, infection after tixagevimab/cilgavimab injection had a lower antibody titer at T0EV compared to KTRs who were not infected with SARS-CoV-2. Cut-off for positive test was defined according to the manufacturers’ instructions as a titer ≥ 0.8 U/mL

Next, we examined the anti-receptor binding domain titer before (T0EV) AZD7442 administration. Although not statistically significant, the anti-receptor binding domain titer at T0EV was lower among infected patients compared to KTRs who did not develop infection (Fig. 3).

**Fig. 4:**
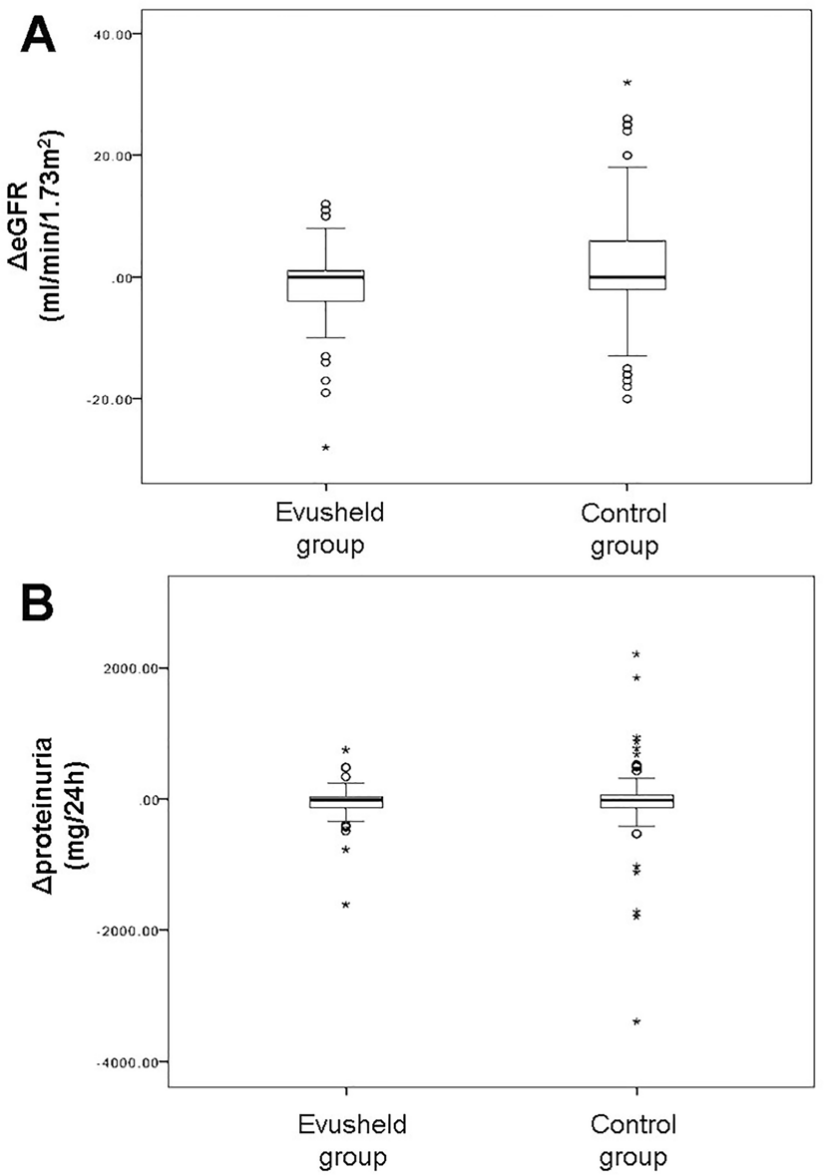
Renal safety of tixagevimab/cilgavimab for pre-exposure prophylaxis in kidney transplant recipients. **A** ΔeGFR (mL/min/1.73 m^2^) before and after at least 1 week from tixagevimab/cilgavimab or fourth vaccine dose administration **B** Δproteinuria (mg/24 h) before and after at least one week from tixagevimab/cilgavimab or fourth vaccine dose administration. The difference was not statistically significant

Finally, in order to evaluate the renal safety of tixagevimab/cilgavimab in KTRs, we analyzed eGFR (mL/min/1.73 m^2^) and proteinuria (mg/24 h) before and after at least one week from the administration of AZD7442. Likewise, we assessed eGFR and proteinuria before and after the administration of the fourth dose of a BNT162b2 mRNA vaccine in the control group. Although not statistically significant, no difference was found (Fig. 4) in eGFR between the AZD7442 group (before 60 ± 5.8, after 62 ± 6.2; *p* = 0.08) and the control group (before 59 ± 9.8, after 60 ± 2; *p* = 0.07). As regards proteinuria, we observed no differences between the AZD7442 group (before 628 ± 50 and after 605 ± 70; *p* = 0.09) and the control group (before 580 ± 60, after 610 ± 20; *p* = 0.08). No other serious adverse events were documented.

## Discussion

Globally, the COVID-19 pandemic has resulted in an increased number of deaths, with differences in the fatality ratio (the number of deaths divided by the number of confirmed cases) among countries due to differences in the number of people tested, demographics and healthcare systems. Moreover, the evolution of SARS-CoV-2 and the emergence of new variants of concern led to changes in clinical presentation and severity of COVID-19 disease. In a recent meta-analysis, Kim K. et al. [12] showed a lower case fatality rate during the Omicron period than that of the COVID-19 Delta epidemic phase. In addition, underlying medical conditions, demographics and exposure factors are associated with a higher risk of severe disease and adverse outcome in COVID-19 [13]. In particular, immunocompromised patients are more likely to develop severe illness even after vaccination because they are unable to present an effective humoral response against SARS-CoV-2 [14, 15]. Solid organ transplant recipients infected with SARS-CoV-2 are at increased risk of morbidity and mortality due to immunosuppression and comorbidities. Nonetheless, the Omicron variant has been associated with less severe disease than the alpha and delta SARS-CoV-2 variants [16], Solid organ transplant recipients remain at high risk for worse outcome than immunocompetent hosts [17–19]. Since the breakthrough of the pandemic, vaccines have represented the greatest hope to control the spread of COVID-19 infection, but some studies have shown that in transplant patients, mRNA vaccines result in poorer neutralizing response against the Omicron variant as compared to the wild-type and delta variants [20, 21]. These data underline the importance of alternative strategies to protect high-risk populations against severe forms of infection caused by SARS-CoV-2 emerging variants of concerns.

Monoclonal antibodies, including casirivimab/imdevimab and tixagevimab/cilgavimab, have been considered promising prophylactic and therapeutic options in vulnerable patients with weak immunological response despite optimal vaccination course [22, 23]. Al Jurdi et al., showed a lower incidence of SARS-CoV-2 breakthrough in solid organ transplant recipients (kidney, liver, lung and multiorgan transplant recipients) who received pre-exposure prophylaxis, but also a lower rate of hospitalizations and deaths in comparison with a control group of solid organ transplant recipients matched for number of vaccines, without significant change in kidney function [24]. Although this study showed similar results as in our study, it was conducted when the B.1.1.529, BA.2, and BA.2.12.1 Omicron lineages were most prevalent in Massachusetts and the results cannot be generalized to BA.4 and BA.5. Our study was conducted in a period in which BA.4 and BA.5 became predominant in Italy.

Nevertheless, initial enthusiasm on the efficacy of these treatments has been curbed by several studies showing breakthrough SARS-CoV-2 infection in patients receiving preventive treatment with anti-spike monoclonal antibodies [25]. Benotmane et al. showed that pre-exposure prophylaxis with tixagevimab/cilgavimab 150/150 mg did not protect KTRs against infection from certain Omicron sublineages such as BA.1, BA.1.1 and BA.2. Remarkably, patients developed symptomatic COVID-19 after pre-exposure prophylaxis, with hospitalizations and deaths [26]. These results were in accordance with those that the same authors observed in a smaller cohort [10]. Conflicting data with our results may be related to the different circulating Omicron sublineages (BA.2, BA.4, BA.5) in the period in which our study was conducted.

Data regarding the safety and efficacy of pre-exposure prophylaxis with neutralizing monoclonal antibodies in solid organ transplant recipients are scarce since immunocompromised patients are often excluded from phase III trials.

In this study we compared for the first time, to the best of our knowledge, the occurrence and severity of SARS-CoV-2 infection in a group of KTRs receiving pre-exposure prophylaxis with tixagevimab/cilagvimab (AZD7442 group) and a group of KTRs vaccinated with four doses of BNT162b2 mRNA vaccine (control group). Remarkably, we found that the prevalence of SARS-CoV-2 infection among patients treated with tixagevimab/cilgavimab 150/150 mg, was significantly lower than in the control group (13.3%; 13/98 vs 34.2%; 53/155). Moreover, KTRs fully vaccinated with four doses of BNT162b2 mRNA vaccine, showed a higher proportion of symptomatic infections (35.8% with mild symptoms and 9.4% with moderate/severe symptoms). According to the most recent evidence in the literature, the majority of KTRs in the AZD7442 group had a favorable outcome after COVID-19 infection, showing the benefit of additional protection for immunocompromised non-responders or low-vaccine responders [27–29]. As shown by Kaminski et al. in a large cohort of KTRs with low or no response to vaccine, a single dose of tixagevimab/cilgavimab 150/150 mg was effective in preventing severe disease and COVID-19-related hospitalizations [27]. At the same time, Bertrand et al. reported that KTRs who received tixagevimab/cilgavimab showed similar outcomes to vaccinated patients but lower rates of infection in comparison to KTRs with low response to vaccine and not treated with pre-exposure prophylaxis [29].

The worse outcome of COVID-19 in the 4-mRNA vaccine dose group may also be due to the higher proportion of patients with HBV and HCV-related chronic liver disease and malignancies in this group. Immune dysregulation associated with chronic liver disease led to an increased risk of severe disease and complications from COVID-19 [30, 31]. Elevated liver enzymes also seem to correlate with the severity of, and mortality from, SARS-CoV-2 infection [32]. Patients with a history of cancer have higher rates of mortality and adverse outcome compared to the general population, and a higher occurrence of complications related to COVID-19 [33–35]. The poorer outcome of COVID-19 in this population is related to cancer subtype, age, and comorbidities but it does not seem to be related to recent systemic anticancer treatments [36].

Another interesting finding in our study was that the majority of patients who did not develop SARS-CoV-2 infection after the administration of tixagevimab/cilgavimab had a higher anti-receptor binding domain titer at T0EV. Although not statistically significant, this could possibly be attributed to an additional effect of vaccination and administration of AZD7442. Further studies are needed to confirm this hypothesis.

In our cohort, tixagevimab/cilgavimab was well tolerated and no major side effects were reported. It is of great importance to note that in the PROVENT study, cardiac adverse events including myocardial infarction, arrhythmias and cardiac failure were documented. In addition, this is the first study to evaluate the impact of tixagevimab/cilgavimab on renal function and proteinuria in KTRs. To this end, we assessed the eGFR as well as changes in proteinuria before and after the administration of AZD7442, finding no statistically significant differences. Al Jurdi et al. obtained similar results, showing that tixagevimab/cilgavimab in KTRs did not cause significant changes in serum creatinine levels [24].

Though we showed the efficacy of tixagevimab/cilgavimab 150/150 mg in a large population of KTRs during the Omicron spread, other studies reported that this dose was not sufficient to achieve significant neutralizing activity against the variant sublineages in vivo [10, 37]. Moreover, as shown in pharmacokinetic modeling, the neutralizing activity against Omicron subvariants seems to be maintained for six months. After tixagevimab/cilgavimab administration, a decline in the anti-receptor binding domain titer, associated with a lack of neutralizing effect against the Omicron variant of concern, has been observed in KTRs [38]. While a correlate of protection for the COVID-19 vaccine could be reasonably represented by the antibody titer [39], for monoclonal antibodies it has not yet been identified. Some studies tried to analyze the protective effect of antibodies after the administration of the COVID-19 vaccine or monoclonal antibodies [40], but this comparison has some limitations due to the different mechanisms of protection. Monoclonal antibodies confer passive immunity, while protection induced by vaccination has more complex mechanisms. For this reason, the significance of antibody titer in predicting immunological protection after monoclonal antibody administration is controversial.

In June 2022, the FDA authorized revisions to tixagevimab/cilgavimab dosing, recommending administration with an increased dose of tixagevimab 300 mg and cilgavimab 300 mg. After the FDA revised the tixagevimab/cilgavimab dosing, high risk patients who had already received the lower dose (150/150 mg) should have received an additional dose (150/150 mg), because the higher 600 mg dose (tixagevimab/cilgavimab 300/300 mg) had shown a protective effect against some SARS-CoV-2 subvariants [41, 42] The superior efficacy of a higher dose of tixagevimab/cilgavimab in pre-exposure prophylaxis seems to be confirmed also in solid organ transplant recipients and against certain Omicron sublineages [43].

A strong point of our study lies in the period during which it was carried out. In fact, the most prominent trials were performed during the Alpha and Delta waves, and they did not include solid organ transplant recipients. Thus, data on the efficacy of tixagevimab/cilgavimab on the Omicron variants in KTRs are limited. On the contrary, some evidence proved that Omicron variants are able to escape neutralization by both casirivimab/imdevimab and tixagevimab/cilgavimab [44–46]. During the Omicron period, we showed the efficacy of pre-exposure prophylaxis with tixagevimab/cilgavimab 150/150 mg in preventing SARS-CoV-2 infection and severe disease without significant adverse events or changes in renal function in our cohort of KTRs, including those transplanted less than one year before. Further data are required to confirm the preliminary findings showing that an increased dose of tixagevimab/cilgavimab 300/300 mg could improve its protective efficacy against SARS-CoV-2.

This study was conducted during a period in which the Omicron variant was the most prevalent in Italy, in particular BA.2 was predominant in March, April and May, while BA.4 and BA.5 became predominant from the beginning of June 2022. This is of importance as the protective effects of AZD7442 against the BA.4 and BA.5 sublineages have not yet been demonstrated.

In conclusion, our data, together with recent reports in the literature, argue for the extensive use of early pre-exposure prophylaxis with tixagevimab/cilgavimab among immunocompromised patients, to protect them from infection or severe COVID-19.

Additional studies are needed to confirm its efficacy against the Omicron sublineages and new emerging variants, and to define whether a higher dose or additional administrations of tixagevimab/cilgavimab could improve its efficacy in this immunocompromised population.

## Data Availability

The datasets generated and analysed during the current study are available from the corresponding author on reasonable request.
